# PTML models of self assembled ligand free nanoparticle catalysts for cross coupling reactions

**DOI:** 10.1038/s41598-025-14080-2

**Published:** 2025-08-14

**Authors:** Andrea Ruiz-Escudero, Zuriñe Serna-Burgos, Sonia Arrasate, Humberto González-Díaz

**Affiliations:** 1 Department of Computer Science and Information Technologies, Faculty of Computer Science, University of A Coruña, Campus Elviña S/N, 15071 A Coruña, Spain; 2 IKERDATA S.L., ZITEK, University of Basque Country UPVEHU, Rectorate Building, 48940 Leioa, Spain; 3 Department of Organic and Inorganic Chemistry, Faculty of Science and Technology, University of The Basque Country (UPV/EHU), P.O. Box 644, 48080 Bilbao, Spain; 4 IKERBASQUE, Basque Foundation for Science, 48011 Bilbao, Spain

**Keywords:** PTML, Cheminformatics, Ligand-free, Self-assembly, Nanoparticles, Cross-coupling reaction, Catalysts, Catalysis, Heterogeneous catalysis, Cheminformatics, Nanoparticles

## Abstract

Cross-coupling reactions have transformed the synthesis of complex and valuable compounds used in pharmaceuticals, materials science, and chemical synthesis. Transition metal nanoparticle (NP) catalysts represent a promising strategy within this field, but their behavior and efficiency continue under investigation. The use of computational models enables rapid design, optimization, and understanding of the behavior of these molecules, thereby reducing the costs and time. In this study, the perturbation theory and machine learning (PTML) approach was used to construct a predictive model for estimating yield after multiple reuses (up to 10) of self-assembled Au- or glass-supported transition metal NP catalysts under ligand-free conditions and diverse cross-coupling reactions. The studied reactions include Suzuki–Miyaura, Kumada, Negishi, Buchwald-Hartwig, C(sp^2^)- and C(sp^3^)-H functionalization, and double carbonylation. A comprehensive dataset was built, and multiple linear regression (MLR) and artificial neural network (ANN) models were built and compared. The best MLR model achieved MAE = 7.4% and RMSE = 12.2% on the test set, demonstrating robust performance for yield prediction. Among the ANN models, MLP (9:9-20-9-1:1) and RBF (9:9-70-1:1) regression models showed similar results, with test MAE of 5.9% and 5.8% respectively, and both showed test RMSE of 9.8%. MLP (9:9-20-18-1:1) classification model showed high precision (97.0%) and recall (93.8%), effectively distinguishing high- and low-yielding reactions. These results highlight the potential of PTML-based models to guide catalyst and reaction condition selection, optimize catalytic systems, and minimize synthesis costs and environmental impact.

**Supplementary Information:**

The online version contains supplementary material available at 10.1038/s41598-025-14080-2.

## Introduction

The use of transition metal nanoparticle (NP) catalysts for the synthesis of fine chemical products, particularly in C–C and C-heteroatom bond-forming reactions, has attracted increasing interest in recent years^1^. These cross-coupling reactions play important roles in the synthesis of a wide variety of compounds used in various industrial and medical fields^2^. This is highly important in the field of organic synthesis because the use of these reactions as versatile, efficient, and selective methods enables the formation of complex molecules with a high degree of specificity^3^. In this work, the reactions studied are Suzuki–Miyaura^4^, Buchwald-Hartwig^5^, Kumada^6^, Negishi^7^, C(sp^2^)- and C(sp^3^)-H functionalization^8,9^, and double carbonylation^10^ catalyzed by transition metal NPs.

One of the main advantages of using metal NPs as catalysts is their high surface-to-volume ratio, which allows for more efficient binding between reactants, resulting in more efficient catalytic activity^11^ Among transition metals, Pd is frequently utilized because of its mild reaction conditions and simple, efficient, and economical protocols. Its versatility makes it a highly desirable metal for synthesizing a wide range of compounds, including highly functionalized molecules, medically important intermediates, drugs, and agrochemical products^12^.

However, the quest for sustainable catalysis has led to research on metals other than Pd. Other metals, such as Ni and Fe, have also been studied as Pd alternatives. These metal NPs are promising substitutes because they are relatively abundant, affordable, and environmentally friendly. Their enhanced recyclability, further reduces their environmental footprint^13^.

Our research focuses on self-assembled metal NPs supported on Au (SAM) and different glass substrates (SGlM). To optimize the catalytic performance, these supporting materials are cleaned with piranha solutions, which remove impurities and incorporate sulfur atoms onto the surface, increasing metal adhesion and cohesion, thereby reducing leaching^14^.

In an effort to broaden the range of sustainable nanocatalytic systems, our study includes a wide range of recycling trials with SAPd(0), SARu(0), SANi(0), and SAFe(II), all of which exhibit exceptional attributes: minimal leaching and high recyclability, even under ligand-free conditions^15,16^.

The design and optimization of these catalysts can be very complex^17^; hence, computational models play a crucial role^18,19^. These models enable the establishment of a connection between the structure of a molecule and its reactivity through the use of parameter sets or descriptors^20,21^. Several models, such as explainable ML techniques or linear regression, provide a better understanding of the behavior and properties of catalysts^22^. Computational models have become essential tools in chemistry, enabling scientists to achieve more precise predictions of chemical behaviors. To date, only a limited number of ML approaches have focused on predicting the performance of multiple cross-coupling reactions within a single framework. Although there are some pioneering efforts to include multi-reaction models^23–25^, studies focusing on ML models trained and validated across multiple, distinct cross-coupling reaction types remain limited. Most models in the literature tend to focus on isolated aspects, such as individual reactions^26,27^ or a single transition metal catalyst^28^, and often do not integrate a comprehensive set of reaction conditions, thereby neglecting the influence of conditions on reaction outcomes.

A commonly used software for generating descriptors is the DRAGON system, which provides a variety of molecular descriptors derived from different molecular representations^29^. The use of computational models in catalyst design and optimization offers several advantages, such as virtual experimentation, a reduction in physical experimentation costs and time, prediction of behavior and properties, and optimization^30^.

In contrast, our work introduces a completely different approach. Using the PTML technique, we have developed a model that not only incorporates all relevant reaction conditions for different cross-coupling reactions, but also introduces the novel capability of predicting reaction yields after the reuse of diverse catalysts. This represents a significant advancement focused on the sustainability and reusability of cross-coupling reactions. Perturbation theory and machine learning (PTML) is an innovative approach that addresses the challenges of selecting appropriate molecular descriptors and developing complex predictive models. PTML combines perturbation theory (PT) and machine learning (ML) techniques to address these issues^31^. PT aims to find a solution to an unknown problem that is comparable to a known solution, whereas ML techniques aid in the selection of molecular descriptors and the creation of predictive models. The combination of these two techniques considers not only the molecular descriptors of a compound but also the difference with respect to the average formed by compounds under similar conditions. These differences are known as moving PT operators (PTOs) or deviations with respect to the moving average^32^. This PTML approach is highly versatile, as it can be applied across various statistical and ML methods, including multiple linear regression (MLR) or linear discriminant analysis (LDA)^33^, or through nonlinear models, such as artificial neural networks (ANNs)^34^. These methods are especially useful for capturing the complexities of ligand-free cross-coupling reactions, in which the reactivity and stability of the catalysts present additional challenges in the absence of stabilizing ligands.

**Fig. 1:**
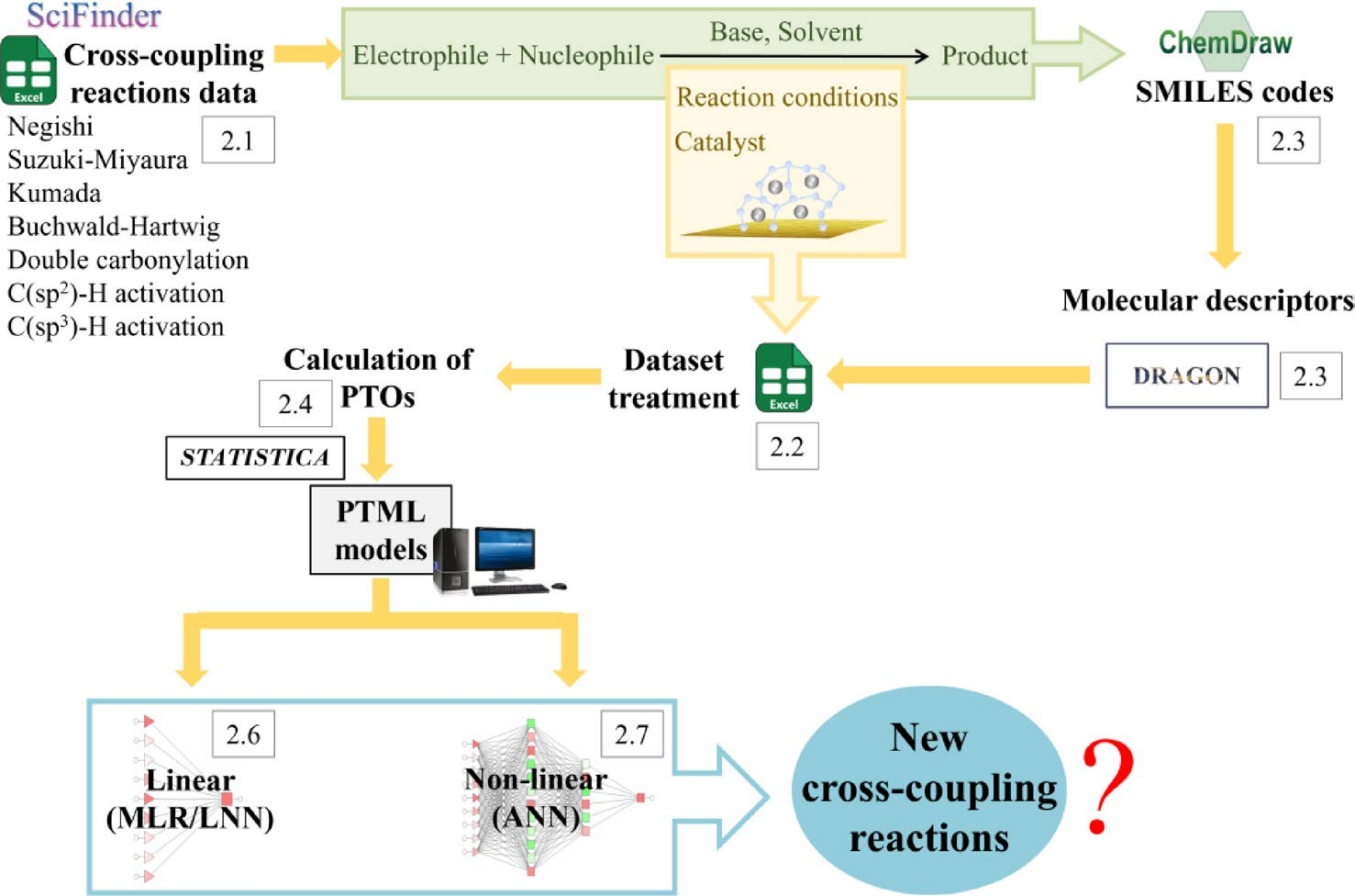
PTML workflow used herein.

The main objective of this work was to design a PTML model capable of accurately predicting reaction yields after multiple reuse cycles (up to 10) of various transition metal NP catalysts under ligand-free conditions. The aim was to establish a rational design for forecasting the optimal characteristics of cross-coupling reactions, enabling chemists to selectively identify the most suitable SAM or SGlM catalyst for maximal efficiency and reusability while minimizing the environmental impact. Figure 1 shows the workflow of the study.

## Materials and methods

### Dataset of cross-coupling reactions catalyzed by metal nanoparticles

In the present work, over a thousand different cross-coupling reactions were manually compiled from more than 100 peer-reviewed publications. The most relevant papers were authored by M. Arisawa, who reviewed and discussed the most significant reactions described in these articles^15^. The primary focus was on analysing the yield parameter Yld(%)_n_ as a function of the number of catalyst reuse cycles (n), with diverse NP systems reused up to 10 times. All the data sources used to compile the dataset can be found in the Supporting Information file SI01.xlsx.

This study focused on the catalytic activity of Pd, Fe, Ni, and Ru NP systems in various reaction types, including Suzuki–Miyaura, Kumada, Negishi, Buchwald-Hartwig, C(sp^2^)- and C(sp^3^)-H functionalization, and double carbonylation. These NPs are supported on Au or glass materials, including different gold structures (gold mesh, gold foil, and gold(111)/mica) and various glass types (alkaline-free glass, quartz glass, white glass, and blue glass), to evaluate their catalytic activity under ligand-free conditions.

The raw dataset was built considering as many reaction properties as possible to account for all the different aspects that could play a crucial role in catalytic success. This includes both catalytic system properties (synthesis procedure, support, NP size, etc*.*) and reaction conditions (reagent quantities, time, temperature, etc.).

Among the reactions, several include two steps; therefore, this variable was also considered by separating the data from step 1 and step 2 into different columns to capture all the details. Additionally, as the nucleophile was added in different steps in diverse reactions, to consider this variable, a value of 0 was assigned for intramolecular reactions, 1 for nucleophile addition in step 1, 2 for step 2, and 3 for both steps. These functional variables were labelled V_k_. For the complete list and details of V_k,_ see Table S1 in the Supporting Information file SI02.

### Data preprocessing and arrangement

In the catalyst data, several reactions lacked information on the quantities of metal adsorbed and released from the support. To ensure the quality and completeness of the dataset and maintain the integrity of each variable, missing data were obtained by using the mean values of known amounts of catalysts with the same metal, support, synthesis procedure, and reuse time. This approach prevents data loss by addressing missing values in a way that accounts for the nature of each variable, thereby minimizing the potential impact of bias^35^ All the calculated values for missing data are reported in the Excel file in purple, and the extracted data from the literature are reported in green (Supporting Information SI01.xlsx). In addition, the dataset was formatted in the Simple User-Friendly Reaction Format (SURF)^36^.

### Calculation of the molecular descriptors

Once the dataset was collected and preprocessed, the next step was to calculate the molecular descriptors (D_k_) of each chemical compound. For this purpose, the SMILES codes were obtained via ChemDraw and subsequently used to calculate molecular descriptors with DRAGON software (v 5.3)^29^. It was possible to calculate up to 1664 different molecular descriptors. However, to prevent potential overfitting^38,39^, a selection of 29 descriptors was chosen based on their potential impact on the reaction properties of the compounds, including constitutional descriptors, functional group counts, and molecular properties. For the complete descriptions, see Table S2 in the Supporting Information file SI02.

The most informative descriptors are those that vary with subtle modifications, such as changes in structure or quantity. Therefore, several D_k_ and V_k_ values of the catalyst, electrophile, nucleophile solvent, and base were multiplied by their corresponding quantities, as presented in Table S3 (Supporting Information File SI02). For the reagents added in both steps, the total quantity was considered.

### PTO calculation

**Fig. 2:**
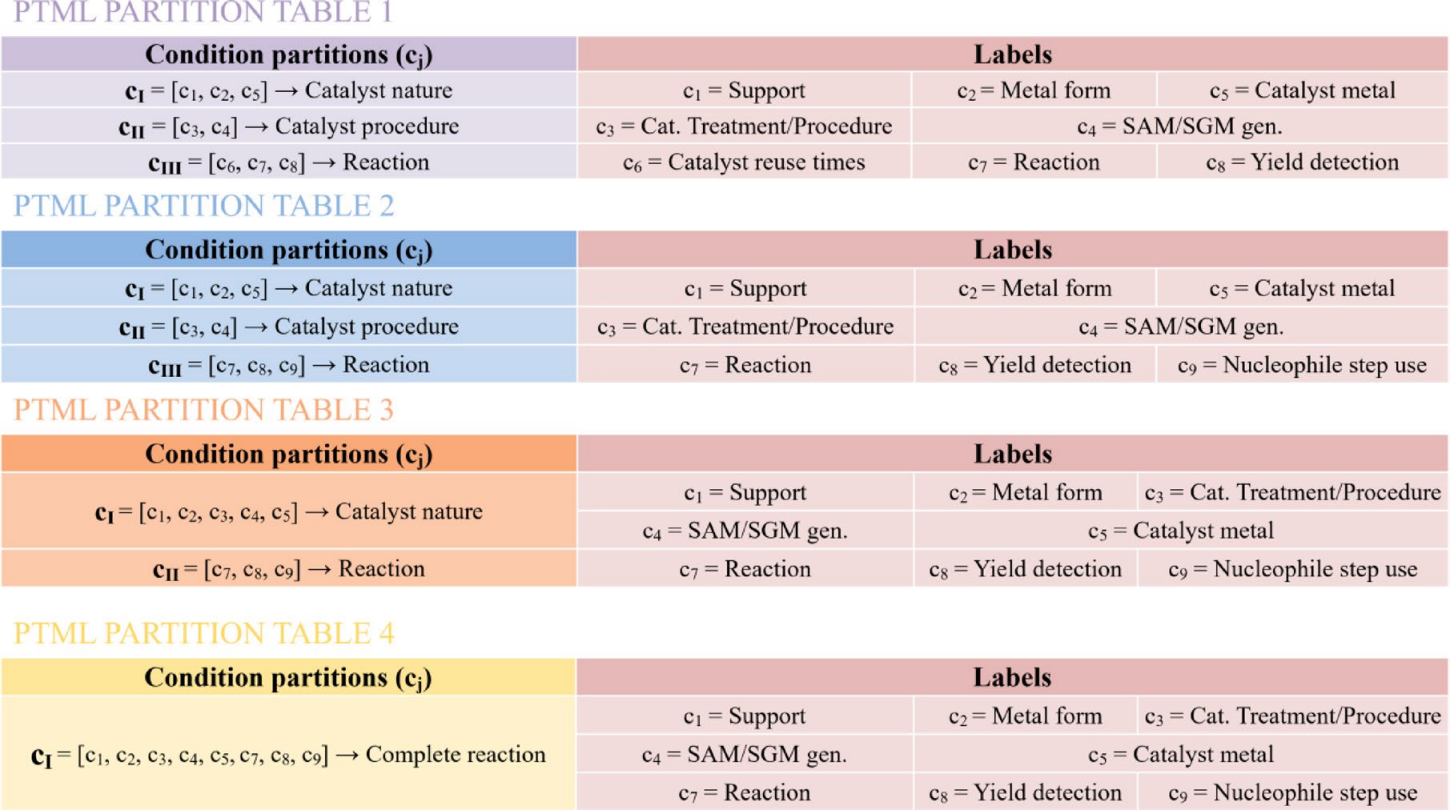
Description of the partitions and labels of the four condition partition tables.

To calculate PT operators (PTOs), nine qualitative variables were selected as reaction condition variables (c_j_) (detailed in Table S4 in the Supporting Information file SI02). These c_j_ variables can be grouped in many ways to generate different partitions or classifications of each reaction trial to have diverse perspectives and compare the model’s output depending on the different aspects of the reaction. To achieve this, four different condition partitioning tables were created (Fig. 2).

Initially, a condition partition comprising three distinct partitions denoted as c_I_, c_II_, and c_III_ was created. Partition c_I_ included information related to catalyst’s nature, support type, form, and metal. In contrast, the c_II_ partition comprised details regarding the catalyst preparation process, support treatment and generation of the procedure employed to assemble the metal in the support.

Finally, partition c_III_ encompassed reaction characteristics, catalyst reuse frequency, reaction type, and yield detection method. Since the output attribute includes catalyst reuse time, c_6_ was replaced by the variable indicating the nucleophile addition step, without altering the overall partitioning scheme. A third condition partition was created, consisting of two partitions labelled cI and c_II_. The c_I_ partition included everything related to the catalyst, and the c_II_ partition included the previously mentioned conditions related to the reaction. Finally, a condition partition was created that included all the previous properties, covering all the reaction characteristics.

The moving averages of each partition were calculated by averaging the values of D_k_ for each molecule (m_i_) and V_k_ for each reaction (r_i_) within each condition partition (c_j_), denoted as D_k_(m_i_)〉c_j_ and 〈V_k_(r_i_)〉c_j_. The PTOs for the molecular descriptors (ΔD_k_) and variables (ΔV_k_) were then calculated as follows:

1 $${\Delta D}_{k}\left({m}_{i},{{\varvec{c}}}_{j}\right)= {D}_{k}\left({m}_{i}\right)- {\langle {D}_{k}({m}_{i})\rangle }_{{{\varvec{c}}}_{j}}$$

2 $${\Delta V}_{k}\left({r}_{i},{{\varvec{c}}}_{j}\right)= {V}_{k}({r}_{i})- {\langle {V}_{k}({r}_{i})\rangle }_{{{\varvec{c}}}_{j}}$$

These PTOs measure the difference between the quantitative value of each reaction and the average value of the corresponding group. This helps to highlight variances that may be important for modelling the reactions.

### PTML model training and validation

To prepare for model training and validation, a random stratified sampling method was used, ensuring that the selected training and validation sets were representative of the entire dataset ^33^. To ensure proper stratification, the dataset was sorted by reaction type and from highest to lowest performance. Each reaction was labelled as “t” (training) or “v” (validation), with 75% of the data for training and 25% reserved for validation. This was achieved by selecting every fourth reaction as a validation sample, the remaining three assigned to training. This approach ensured a balanced distribution of data across the sets, supporting robust model development and reliable outcomes.

### PTML models

This section details the training and evaluation of predictive models using MLR and ANN models. The overall workflow for building these models is shown in Figure SI1 (Supporting Information file SI02). The figure illustrates the workflow, from data selection to validation, and the final model. The figure also shows the section numbers where each step is described.

#### PTML-MLR linear models

For the PTML linear models, the MLR algorithm was used, and the general equation was applied as follows:

3 $${{\varvec{f}}\left({{\varvec{v}}}_{{\varvec{i}}{\varvec{j}}}\right)}_{{\varvec{c}}{\varvec{a}}{\varvec{l}}{\varvec{c}}}={\mathbf{a}}_{0}+{\mathbf{a}}_{1}\cdot {{\varvec{f}}\left({\mathbf{v}}_{\mathbf{i}\mathbf{j}}\right)}_{{\varvec{r}}{\varvec{e}}{\varvec{f}}}+\sum_{{\varvec{i}}=1,{\varvec{j}}=1,{\varvec{k}}=1}^{{{\varvec{i}}}_{{\varvec{m}}{\varvec{a}}{\varvec{x}}},{{\varvec{j}}}_{{\varvec{m}}{\varvec{a}}{\varvec{x}}}{,{\varvec{k}}}_{{\varvec{m}}{\varvec{a}}{\varvec{x}}}}\Delta {\mathbf{D}}_{{\varvec{k}}}\left({{\varvec{m}}}_{{\varvec{i}}},{{\varvec{c}}}_{{\varvec{j}}}\right)\bullet {{\varvec{b}}}_{{\varvec{k}}{\varvec{j}}}+\sum_{{\varvec{i}}=1,{\varvec{j}}=1,{\varvec{k}}=1}^{{{\varvec{i}}}_{{\varvec{m}}{\varvec{a}}{\varvec{x}}},{{\varvec{j}}}_{{\varvec{m}}{\varvec{a}}{\varvec{x}}}{,{\varvec{k}}}_{{\varvec{m}}{\varvec{a}}{\varvec{x}}}}\Delta {\mathbf{V}}_{{\varvec{k}}}\left({{\varvec{r}}}_{{\varvec{i}}},{{\varvec{c}}}_{{\varvec{j}}}\right)\bullet {{\varvec{b}}}_{{\varvec{k}}{\varvec{j}}}$$

The output property function of a reaction, f(v_ij_)_calc_, can be calculated via a reference function, f(v_ij_)_ref_, considering the effects of the moving averages, ΔD_k_ and ΔV_k_, which refer to a specific molecule (m_i_) and reaction (r_i_) under conditions c_j_, and the known constants (a_0_, a_1_, and b_kj_). The reference function was calculated as the average of each set considering the output property (c_0_) as the conditioner.

4 $$f{\left({v}_{ij}\right)}_{ref}= {\langle Yield(\text{\%})\rangle }_{{{\varvec{c}}}_{{\varvec{j}}}}$$

STATISTICA 6.0 software was used to create the models.^37,40^ First, the forward stepwise (FSW) procedure was used to automatically select the input variables, and a maximum of 10 steps were chosen. Subsequently, expert-guided selection (EGS) was performed and combined with the FWS procedure. In EGS, important features that were previously absent but had a high impact on the reaction, such as the employed reagents, were selected. Prior to model construction, outlier detection was performed via cross-validation. Afterwards, the models were built with the training data and tested with the validation data.

#### PTML-ANN nonlinear models

The correlations between the descriptive and continuous variables and the output yield can be complex and nonlinear in cross-coupling reactions. This complexity could be addressed by employing artificial neural network (ANN) models. To build the nonlinear models, the first step included all the variables from the previously selected PTML condition partition dataset.

In addition, the models were trained with the most relevant variables identified from the best MLR model. This reduces the number of input variables, simplifying and accelerating the training of these models. It also improves the interpretability of the model by focusing on the most relevant variables and discarding the less influential ones. Additionally, a comparison can be made between the best model obtained via MLR and those obtained via the ANN. To perform these models, the training and validation subsets were kept as previously stated (75% training, 25% validation). The network architectures tested included linear neural network (LNN), multilayer perceptron (MLP), generalized regression neural network (GRNN), and radial basis function (RBF). The minimum number of hidden units was 1 for all of them, and the maximum was 300 for the RBF and 20 for the MLP.

#### PTML-ANN classification models

In addition to regression models, classification models can also be very useful when the goal is to classify the reactions as successful or unsuccessful based on their performance rather than when specific values are predicted. For these classification models, the output data were calculated by considering the average yield value of the full dataset (79%). Reactions with yields above 79% were classified as desired (1), while those with yields of 79% or less were classified as undesirable (0). In this way, the model, instead of giving a specific value as in regression, would help discriminate whether the reaction is considered to have good or bad yields.

The input reference values were calculated as the probability of being 1 by averaging the output classifier values for each reusability time (n) in each Yld(%)_n._ The models were built via the same methodology as the PTML-ANN nonlinear models.

## Results and discussion

### PTML-MLR linear models

The MLR models initially constructed using the FWS procedure, limited to 10 steps. This procedure aimed to identify variables that have a high influence to maintain the model’s simplicity. However, there were significant factors that were not included in the models after the FWS procedure, such as the effect of the reagents or the temperature of the reaction. Therefore, an EGS was carried out. This involved retaining FWS-selected variables and manually incorporating additional variables and their partitions.

First, leave-one-out cross-validation was conducted to ensure that each data point contributed independently ^26^. The model was then built from the training data and tested on the validation data. Using the developed MLR equations, the predicted yield values were calculated, and the corresponding relative errors were then computed. This dual validation approach provided a more comprehensive view of the model’s robustness and reliability.

The statistical results of the models for each PTML partition table can be found in Table S5 in the Supporting Information file SI02, along with the variables that included the models, in addition to the intercept and the reference function, < Yld(%)c_0_ > .

The leave-one-out cross-validation and the results of the relative errors showed that the data points of the double carbonylation reactions produced high errors. This suggests that, compared with other reaction types, double carbonylation reactions may involve more unpredictable complex behavior. Factors such as temperature, reagents or other possible reaction mechanisms that may differ from the other reaction types could contribute to these errors.

To visualize the contributions of these challenging data points, the PTML 3 model (Eq. 5) was employed to plot the observed versus predicted values for both the training and test sets (for detailed information, see ESI SI01, Table S3). The model was obtained through 851 training points, with an R value of 0.8181(*p* < 0.05). In addition, MAE and RMSE values were calculated as 9.49% and 15.35% for the training set, and 9.47% and 15.58% for the test set, respectively.

5 $$\begin{aligned} {\text{Yld}}\left( \% \right)_{n} = & 2.0103 + 0.9749 \cdot {\text{Yield}}\left( \% \right)_{c0} \\ & + 0.0001 \cdot \Delta ({\text{D}}_{2} {\text{V}}_{2} )\left( {pm \times \mu g} \right) + 0.0144 \cdot \Delta {\text{V}}_{31} \left( h \right) \\ & + 0.9899 \cdot \Delta {\text{V}}_{33} + 0.1526 \cdot \Delta ({\text{V}}_{11} {\text{V}}_{13} )\left( {{\text{mmol}}} \right) \\ & - 2.9991 \cdot \Delta [{\text{V}}_{29} ({\text{V}}_{22} + {\text{V}}_{26} )]\left( {{\text{D}} \times {\text{mL}}} \right) \\ & + 14.2967 \cdot \Delta ({\text{E}}_{6} {\text{V}}_{8} )\left( {{\text{D}} \times {\text{mmol}}} \right) \\ & - 14.1591 \cdot \Delta [{\text{D}}_{15} ({\text{V}}_{10 } + {\text{V}}_{35} )] + 1.0950 \cdot \Delta {\text{V}}_{30} \left( {^\circ C} \right) \\ \end{aligned}$$

**Fig. 3:**
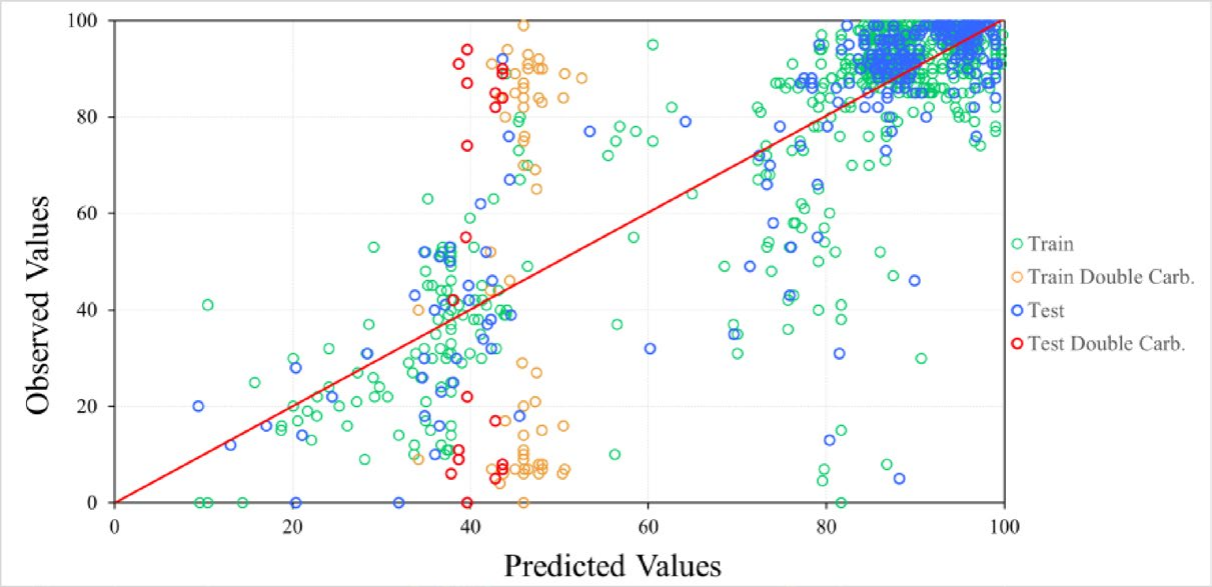
Observed vs. predicted values of the PTML 3 training and testing data.

As shown in Fig. 3, the predicted yields of the double carbonylation reactions exhibit a weak correlation with both the observed yields in the training data (represented in orange) and the validation data (represented in red). This highlights the difficulty in accurately predicting the yields of these reactions.

To explore the global impact of this particular subset on the model’s performance, leave-group-out cross-validation was carried out^41^. This method excluded the double carbonylation subset, providing a clearer assessment of the model’s performance without this particular reaction group. The results are summarized in Table S6 (Supporting Information file SI02). The PTML 3 and PTML 4 partition tables achieved the highest correlation between the observed yield and the predicted yield for the training data. To determine the most suitable model, predictions were made across all partition tables via the validation data. The results of the observed vs. predicted yields of PTML 3 and PTML 4 for the training and test data are shown in Figure SI2.

The resulting correlations for the validation data were 0.8627 and 0.7261 for PTMLs 3 and 4, respectively. These results demonstrate that by removing the double carbonylation set, the model shows better overall generalizability. Compared to models including double carbonylation reactions, PTML 3 showed improved RMSE and MAE: 12.40% and 7.46% for the training set, and 12.24% and 7.39% for the test set, respectively. PTML 4 yielded MAE values of 11.00% (training) and 10.77% (test), and RMSE values of 17.15% (training) and 17.17% (test).

6 $$\begin{aligned} {\text{Yld}}\left( \% \right)_{n} = & 1.7555 + 0.9731 \cdot {\text{Yield}}\left( \% \right)_{c0} \\ & + 0.00003 \cdot \Delta ({\text{D}}_{2} {\text{V}}_{2} )\left( {pm \cdot \mu g} \right) + 0.0381 \cdot \Delta {\text{V}}_{31} \left( h \right) \\ & + 1.0443 \cdot \Delta {\text{V}}_{33} - 0.2962 \cdot \Delta ({\text{V}}_{11} {\text{V}}_{13} )\left( {{\text{mmol}}} \right) \\ & - 2.7938 \cdot \Delta [{\text{V}}_{29} ({\text{V}}_{22} + {\text{V}}_{26} )\left( {{\text{D}} \cdot {\text{mL}}} \right) \\ & + 16.8403 \cdot \Delta ({\text{D}}_{6} {\text{V}}_{8} )\left( {{\text{D}} \cdot {\text{mmol}}} \right) \\ & - 16.5780 \cdot \Delta [{\text{D}}_{15} ({\text{V}}_{10 } + {\text{V}}_{35} )] + 1.0987 \cdot \Delta {\text{V}}_{30} \left( {^\circ C} \right) \\ \end{aligned}$$

Thus, among both partition tables, the model obtained using PTML 3 was selected as the most appropriate (Eq. 6). This model was built with a total of 789 different training points and an R value of 0.8604 (*p* < 0.05).

### PTML-ANN nonlinear models

To build the ANN models, the PTML 3 partition dataset was selected as the basis for the analysis. In one approach, the entire dataset, including all reaction types, was used for both training and validation. In the second approach, the double carbonylation reactions were excluded from the dataset. The results of these models are summarized in Supporting Information files SI02 and Table S7.

The results of the models without the double carbonylation set, similar to the MLR models, enhance the correlation between the observed and predicted data across all four different profiles (LNN, MLP, RBF and GRNN). Like the linear models,

The inclusion of the double carbonylation reactions introduced additional complexity, making it more difficult for the models to achieve optimal predictive accuracy.

Although the models showed acceptable correlations, the software program STATISTICA 6.0 tended to select an excessive number of input variables, which could negatively impact model efficiency and interpretability. Among the ANN models that excluded the double carbonylation reactions, the RBF model showed enhanced correlations for both the training and testing sets, with the fewest variables used as inputs. This makes the RBF model particularly interesting for applications prioritizing simplicity and computational efficiency, while maintaining accuracy. Even so, several variables included in the model do not have a significant effect.

To facilitate the comparison between the ANN and MLR models and to limit the number of variables, the following ANN models were constructed by employing an EGS. The selected variables for these models were derived from the PTML3 MLR model (Eq. (5)). On the one hand, the models were constructed using the double carbonylation subset, while on the other hand, they were constructed without incorporating it.

Table 1a presents the results of the ANN models with all subsets included, while Table 1b provides the results for the models without the double carbonylation subset, along with their respective network illustrations. Comparison of the correlation values (R) for the different models confirms that, as with the MLR and previous ANN models, exclusion of the double carbonylation subset consistently results in higher correlation coefficients and lower RMSE and MAE values.

**Table 1:** Results of the ANN models with EGS variables (PTML 3).

Model*	R (train)	R (test)	MAE (train)	MAE (test)	RMSE (train)	RMSE (test)
	0.795	0.822	9.500	9.539	15.353	15.554
	0.810	0.849	8.385	8.707	14.248	14.560
	0.778	0.790	15.276	15.347	22.494	22.573
	0.783	0.859	7.904	8.482	13.802	15.335
	0.865	0.862	7.383	7.392	12.187	12.252
	0.917	0.915	5.700	5.864	9.715	9.789
	0.915	0.915	5.509	5.761	9.817	9.768
	0.838	0.830	13.261	13.256	19.941	19.894

^a^Models with the complete dataset. ^b^Models without the double carbonylation subset. ^c^The codes are BP = Back Propagation, CG = Conjugate Gradient Descent, SS = Subsample, KM = K-Means (Centre Assignment), KN = K-Nearest Neighbour (Deviation Assignment), and PI = Pseudo-Invert (Linear Least Squares Optimization). *Model designations follow the format A:B-C-D-E:F, where A is the number of input features; B, C, and D are the number of neurons in each hidden layer; and E, F are the number of output neurons.

**Fig. 4:**
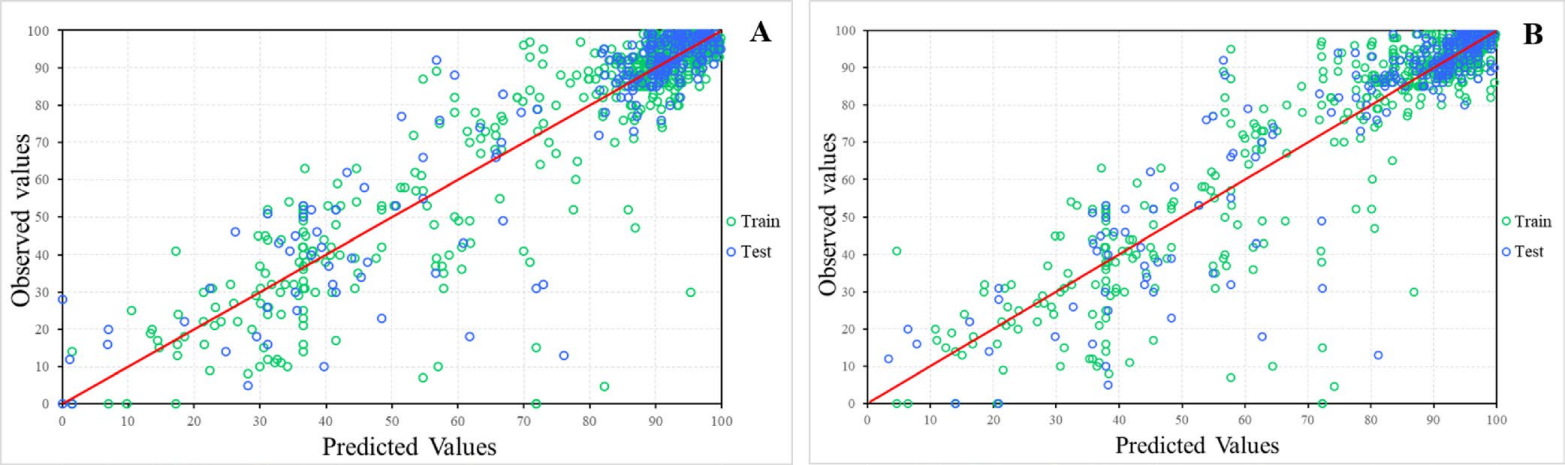
Observed values vs. predicted values of the training and validation data of the MLP (9:9-20-9-1:1) (**A**) and RBF (9:9-70-1:1) (**B**) models.

Among the ANN models, the MLP (9:9-20-9-1:1) and RBF (9:9-70-1:1) models yield high correlation values between the observed yields and the predicted yields. Additionally, these models yield the lowest MAE and RMSE values with minimal differences between the training and testing sets, suggesting robust generalizability and low overall predictive errors. Figure 4 shows the scatter plots of observed vs. predicted yields for these MLP (A) and RBF (B) models, with the green dots representing the training set and the blue dots representing the test set. Given the dataset’s imbalance favouring high yields, the model’s predictions for lower yields remain reasonably accurate, avoiding overestimation.

In addition, given this data distribution, which could cause bias in the analysis, additional evaluations were performed by calculating the MAE and RMSE values for yields below and above 79%—the average yield of the dataset —to provide a more comprehensive assessment of each performance metric.

Both models performed well on high-yield reactions (> 79%), obtaining similar MAE and RMSE low values across the training and validation sets (Table 2). However, when predicting the lower yields (≤ 79%), there is an increase in the error observed for both models. The MLP model yields MAE and RMSE values of 13.63% and 17.97%, respectively, for the validation set and, similarly, RBF values of 13.20% and 17.61%, respectively. These findings suggest that a classification instead of a regression could yield more accurate predictions.

**Table 2:** Training and test MAE and RMSE values of the MLP (9:9–20–9–1:1) and RBF (9:9–70–1:1) models for yields below and above 79%.

Model	Dataset	Yield range (%)	MAE (%)	RMSE (%)
MLP 9:9-20-9-1:1	Training set	> 79	3.77	5.39
		≤ 79	12.39	17.91
	Test set	> 79	3.67	5.46
		≤ 79	13.63	17.97
RBF 9:9-70-1:1	Training set	> 79	3.55	5.54
		≤ 79	12.31	17.99
	Test set	> 79	3.60	5.74
		≤ 79	13.20	17.61

### PTML-ANN classification models

To address the challenges of predicting low-yielding reactions in regression models, due to the unbalanced nature of the dataset, classification models were developed to differentiate reactions into two categories: high performance (> 79%) and low performance (≤ 79%).

The classification models were built using the complete dataset, including the double carbonylation reaction subset. The input values corresponded to those previously selected, specifically the EGS variables from the PTML 3 partition, to compare the performance of the classification model to those of the previous MLP (9:9-20-9-1:1) and RBF (9:9–70–1:1) regression models. Among the tested architectures, the MLP (9:9-20-18-1:1) demonstrated high accuracy and robust generalizability, as shown in Table S8.

The training results show that the model performs exceptionally well in classifying high- and low-performance reaction outcomes. The high precision score (0.98) indicates that nearly all high-yield reactions were correctly identified in high yield. The recall value of 0.94 further demonstrates that the model successfully identifies most high-performance reactions, whereas the F1 score of 0.96 proves the strong balance between precision and recall.

The test results closely aligned with the metrics of the training set, suggesting an enhanced generalization of the model. The minimal decreases in accuracy (from 0.94 to 0.93) and precision (from 0.98 to 0.97) indicate limited overfitting, whereas the recall (0.94) and F1 score (0.96) remain consistent, confirming that the model maintains its predictive performance when applied to unseen data.

**Fig. 5:**
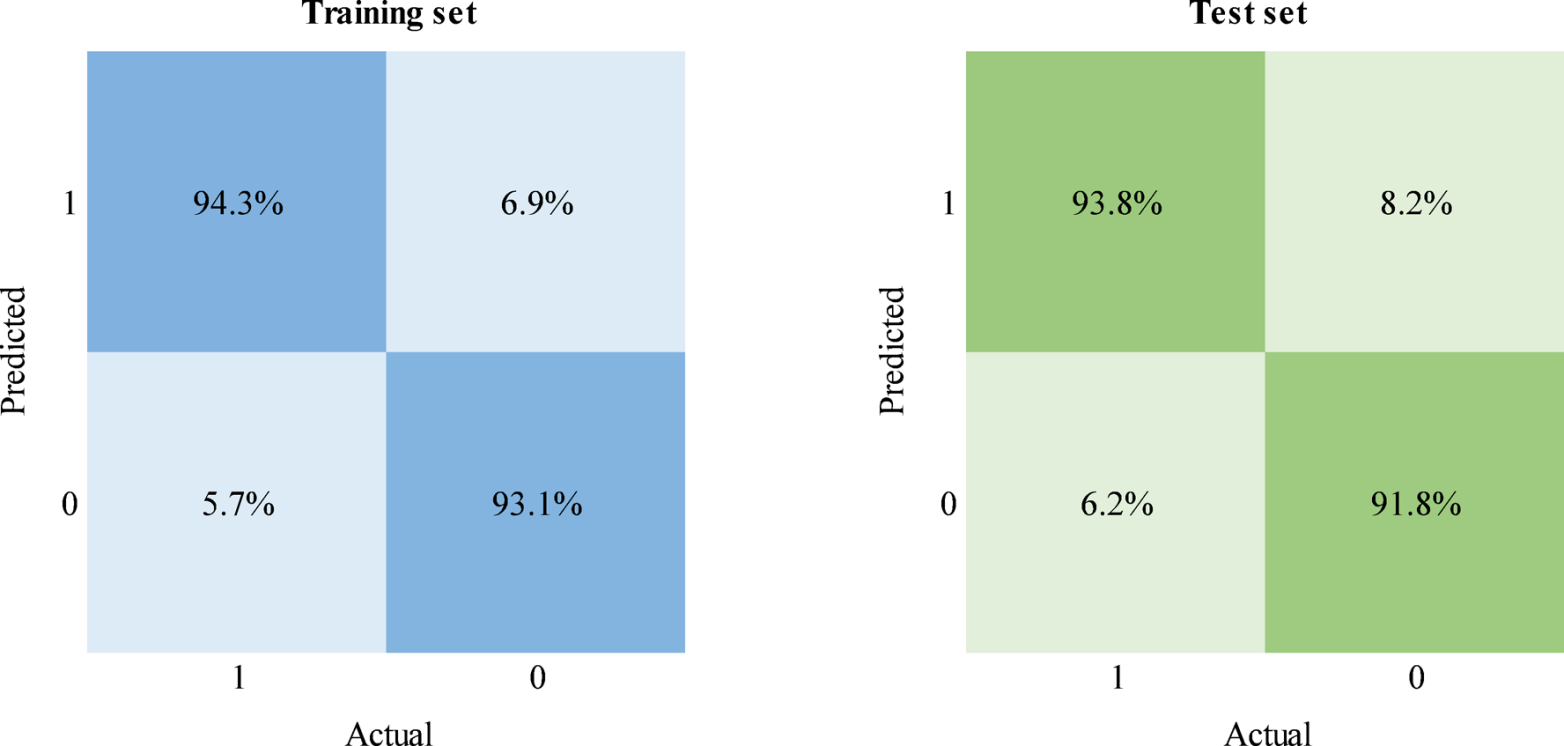
Confusion matrices of the training and test sets with the MLP 9:9-20-18-1:1 classification model.

To further analyse the impact of dataset imbalance, the confusion matrices of the training and test sets were plotted (Fig. 5).

The confusion matrices indicate that the model achieves consistently high true positive rates, with values of 94.3% and 93.8% for the training and test sets, respectively, and consistent true negative rates of 93.8% and 91.8% for training and testing, respectively.

This balanced performance across both datasets highlights the reliability of the model for distinguishing high-performance (> 79%) and low-performance (≤ 79%) outcomes. This finding highlights the model’s ability to learn effectively from complex patterns in diverse reaction conditions, despite dataset imbalance.

**Fig. 6:**
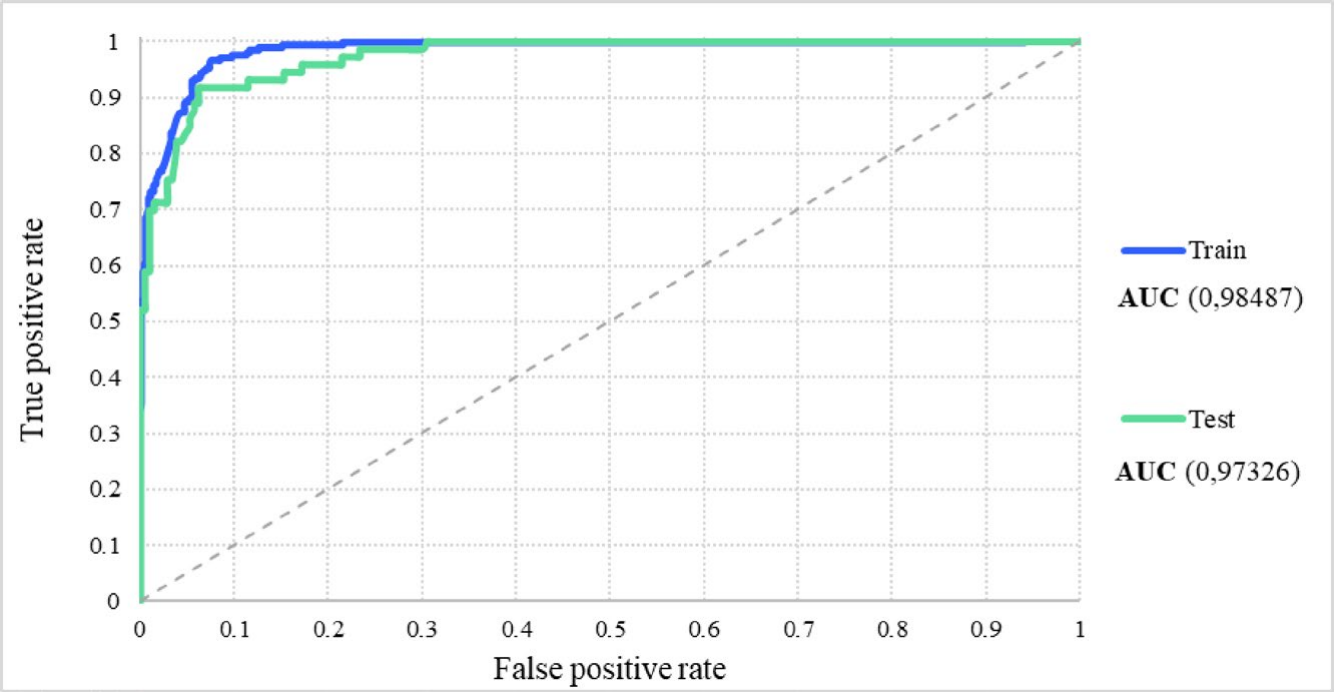
ROC curves for the training and test sets of the MLP (9:9-20-18-1:1) classification model.

Additionally, receiver operating characteristic (ROC) curves were plotted to assess the model’s ability to balance true positive rates against false positive rates across different classification thresholds of the training and test sets (Fig. 6).

The ROC curves demonstrate the model’s excellent discriminative ability, with areas under the curve (AUCs) of 0.98 and 0.97 for the training and test sets, respectively. These values further highlight the model’s effectiveness in distinguishing between high- and low-performance reaction outcomes. Additionally, the minimal difference between the training and test AUC values supports the model’s robustness and minimal overfitting, which is consistent with the previously analysed performance metrics. The nearly perfect AUC scores validate the model’s enhanced performance and its suitability for classifying reaction outcomes.

To provide further insight into the variables influencing the classification model’s prediction decisions, feature importance scores were analyzed (see Figure SI3 in the Supporting Information file SI02). The analysis revealed that the variables related to catalysts had the highest influence on the model’s output. This suggests that the nature and amount of the metal catalyst are critical factors in determining reaction performance in the studied cross-coupling reactions. The catalyst’s reuse cycle is also an important descriptor, highlighting the role of the reusable catalyst’s longevity and stability. This is an interesting outcome because conventional ML studies do not focus on catalyst reusability, and when using a dataset with reaction yields for different catalytic cycles, the model demonstrated that this is a key factor for the predictions. Other variables associated with the base also showed notable importance, reflecting the relevance of the reaction medium in facilitating the transformations. Variables related to the nucleophile, time, and temperature contributed to the model but with lower influence than the catalyst’s nature. Overall, all the variables included in the model had a meaningful contribution to its predictive capability, with none showing negligible influence, which also highlights the relevance of the previously selected descriptors using the EGS.

In the previous regression models, double carbonylation reactions significantly affected the model’s performance, and although excluding this set obtained improved predictive accuracies, this reduced the dataset complexity. However, in this classification model, these reactions did not negatively impact performance, with overall performances of 94% and 93% for the training and test sets, respectively. By prioritizing categorical separation over exact numerical predictions, the classification approach effectively integrates the complete dataset without compromising the generalization capacity.

These results confirm that the MLP (9:9-20-18-1:1) classification model has optimal generalizability across both datasets, which ensures practical applicability in predicting reaction performance.

### Overview of recent ML models for cross-coupling reactions

To contextualize the novelty and performance of our present PTML-based models, Table 3 presents an overview of our findings with various recent ML studies focused on cross-coupling reaction’s yield prediction. The table reports the reported best test metrics (RMSE for regression and accuracy for classification) for each study, according to the different employed featurization methods and model architectures, and the highest predictive performances for regression and/or classification models within each study are highlighted in bold. A wide range of models is included, from the traditional approaches such as random forests (RF), k-nearest neighbours (KNN), linear regression (LR), to state-of-the-art deep learning architectures such as message passing neural networks (MPNNs), residual graph convolutional networks (ResGCN), graph attention networks (GAT and GATv2), graph convolutional networks (GCN), and graph isomorphism networks (GIN), among others. In addition to these architectures, diverse featurization strategies are reported due to their crucial role in the prediction outcomes.

**Table 3:** Overview of ML models for predicting yields in cross-coupling reactions.

Model’s reactions	Catalyst metals	ML architectures	Params	Test values (%)	Featurization method	References
Suzuki–Miyaura, Kumada, Negishi, Buchwald-Hartwig, and C(sp^2^)- and C(sp^3^)-H functionalization	Pd, Fe, Ni, and Ru	PTML-MLR	RMSE	12.24	DRAGON molecular descriptors	This work
		PTML-MLP		**9.79**		
Suzuki–Miyaura, Kumada, Negishi, Buchwald-Hartwig, C(sp^2^)- and C(sp^3^)-H functionalization, and double carbonylation		PTML-MLP	Accuracy	**93.40**		
Suzuki, Sonogashira, Cadiot-Chodkiewicz, Ullmann, and Buchwald-Hartwig	Mn, Fe, Co, Cu, Pd, and Zn	KNN (HTP)	Accuracy	60.00	RDKit FP	23
				**66.00**	DRFP	
				59.00	RXNFP	
		NN	RMSE	14.23	DRFP	
				15.18	RDKit FP	
				20.50	RXNFP	
		RF		**13.56**	DRFP	
				14.33	RDKit FP	
				19.95	RXNFP	
Suzuki, Sonogashira, Cadiot-Chodkiewicz, Ullmann, and Buchwald-Hartwig	Mn, Fe, Co, Cu, Pd, and Zn	MPNN	RMSE	**14.55**	RDKit molecular descriptors	24
		ResGCN		14.62		
		GATv2		15.00		
		GraphSAGE		14.56		
		GCN		16.05		
		GAT		16.18		
		GIN		24.28		
Suzuki	Pd	NN	Accuracy	74.30	OHE	26
				76.40	RDKit FP	
				73.90	DFT	
		RF		73.90	OHE	
				76.80	RDKit FP	
				76.60	DFT	
		xGB		73.70	OHE	
				76.80	RDKit FP	
				**76.90**	DFT	
Buchwald-Hartwig	Pd	LR	RMSE	15.50	Spartan quantum chemical descriptors	27
		KNN		16.30		
		SVM		15.80		
		Bayes GLM		15.50		
		NN		16.90		
		RF		**7.80**		

*PTML*, Perturbation Theory Machine Learning; *MLR*, Multiple Linear Regression; *LR*, Linear Regression; *MLP*, Multi-Layer Perceptron; *KNN*, k-Nearest Neighbours; *HTP*, Hyperparameter Tuning; *NN*, Neural Network; *RF*, Random Forest; *MPNN*, Message Passing Neural Network; *ResGCN*, Residual Graph Convolutional Network; *GAT*, Graph Attention Network; *GraphSAGE*, Graph Sample and Aggregate; *GCN*, Graph Convolutional Network; *GIN*, Graph Isomorphism Network; *SVM*, Support Vector Machine; *Bayes GLM*, Bayesian Generalized Linear Model; *xGB*, Extreme Gradient Boosting; *DRFP*, Differential Reaction Fingerprint; *RXNFP*, Reaction Fingerprint; *OHE*, One-Hot Encoding; *RDKit FP*, Fingerprint generated with RDKit; *DFT*, Density Functional Theory.

Significant values are in [bold]

As shown in Table 3, our PTML-based models demonstrate strong performance within this topic. In particular, the PTML-MLP regression model yielded the lowest RMSE (9.79) among the multi-reaction models. While an RF model reported for the Buchwald-Hartwig reaction using Spartan descriptors shows a slightly lower RMSE (7.80), it is important to note that this model is restricted to a specific reaction class. Unlike our PTML approach, it is not designed to generalize across multiple cross-coupling types and catalysts within a single framework. Furthermore, the PTML-MLP classification model achieved high accuracy (93.40%).

Also, as shown in Table 3, a variety of featurization strategies are represented, including traditional approaches such as classic molecular descriptors (e.g. physicochemical, topological, and structural properties) generated with different software like DRAGON, RDKit, or Spartan. These established features provide interpretable and transparent information in ML models. Even so, recent literature shows a growing trend toward the use of learned or graph-based descriptors to encode molecular and reaction context with greater expressiveness. Notably, Coley et al. demonstrated the predictive power of graph-convolutional neural networks of general chemical reactivity^42^, while Schwaller et al. applied neural sequence-to-sequence models to reaction yield prediction, showcasing the potential of attention-based architecture’s learning power of complex chemical transformations^43^. While these representations have significantly advanced the field, as previously mentioned, they are mainly applied to single reaction types or specific catalytic systems. Also, their black-box nature can limit interpretability and practical adoption in experimental settings. In this context, our work uses descriptors obtained from DRAGON for their proven interpretability and broad molecular coverage, while the flexibility of the PTML approach allows for the future integration of different advanced featurization methods to further enhance predictive power.

## Conclusions

The present study provides a significant contribution to the field of ML applied to chemistry, particularly in predicting different cross-coupling reaction yields within a unified PTML framework. By integrating perturbation theory with machine learning, this approach addresses key limitations of current ML models and offers a practical methodology to optimize ligand-free catalytic systems and reaction conditions, reducing the environmental impacts of traditional trial‒and-error experimentation. The integration of PTML with ANNs, especially MLP regression and classification models, have demonstrated strong potential of advancing catalyst design and reaction optimization.

A key achievement of this research includes the development of a comprehensive dataset covering diverse cross-coupling reactions catalyzed by ligand-free, reusable, self-assembled transition metal NPs. The MLP regression model (9:9-20-9-1:1) demonstrated strong predictive ability across almost all reaction types, except for the double carbonylation subset, where its performance was more limited. Notably, the MLP (9:9-20-18-1:1) classification model also showed excellent predictive performance across Suzuki–Miyaura, Kumada, Negishi, Buchwald-Hartwig, C(sp^2^)- and C(sp^3^)-H functionalization, and double carbonylation reactions. These model’s ability to predict catalyst yields after up to 10 reuses highlights their practical use in selecting optimal catalysts, enabling more cost-effective and environmentally friendly decision-making. Moreover, the robust PTML-MLP (9:9-20-18-1:1) classification model effectively handles the imbalanced dataset and the complex reaction conditions, with enhanced generalizability and reliability in identifying high-yield reactions. This makes both the regression and classification models valuable tools for synthetic optimization.

Future perspectives include refining the feature selection process, exploring ensemble learning techniques, and incorporating more advanced features such as learned or graph-based representations to further improve model performance and generalizability (particularly for challenging cases like double carbonylation reactions). Additionally, to make these models more accessible and practical for the scientific community, we are working on developing a user-friendly, open-source web application that will allow re-searchers to easily apply the models to their own experimental data.

## Supplementary Information

Below is the link to the electronic supplementary material.

Supplementary Material 1

Supplementary Material 2

## Data Availability

The final regression and classification models are publicly available and free of use on GitHub at the following link: https://github.com/Aruize/PTML-Nanocatalysts

## Abbreviations

PTML: Perturbation theory and machine learning
MLR: Multiple linear regression
ANN: Artificial neural network
MLP: Multilayer perceptron
NP: Nanoparticle
SAM: Self-assembled metal in Au
SGlM: Self-assembled metal in glass
PTO: Perturbation operator
LDA: Linear discriminant analysis
MD: Missing data
FWS: Forward stepwise
EGS: Expert-guided selection
LNN: Linear neural network
GRNN: Generalized regression neural network
RBF: Radial basis function
KNN: K-nearest neighbours
NN: Neural network
RF: Random forest
MPNN: Message passing neural network
ResGCN: Residual graph convolutional network
GAT: Graph attention network
GATv2: Graph attention network version 2
GraphSAGE: Graph sample and aggregate
GCN: Graph convolutional network
GIN: Graph isomorphism network
SVM: Support vector machine
Bayes GLM: Bayesian generalized linear model
xGB: Extreme gradient boosting
DRFP: Differential reaction fingerprint
RXNFP: Reaction fingerprint
OHE: One-hot encoding
FP: Fingerprint
DFT: Density functional theory
HTP: Hyperparameter tuning
