# Supplementary material for: PTML models of self assembled ligand free nanoparticle catalysts for cross coupling reactions
Source: Sci Rep. 2025 Aug 14;15:29895. doi: 10.1038/s41598-025-14080-2 (PMC12354725; doi:10.1038/s41598-025-14080-2)
Supplement: Supplementary file 2 — Supplementary Material 2 [file 41598_2025_14080_MOESM2_ESM.pdf]

Supplementary Information

PTML Models of Self Assembled Ligand Free Nanoparticle Catalysts for Cross Coupling Reactions

Andrea Ruiz-Escudero,[a,b] Zuriñe Serna Burgos,[c] Sonia Arrasate,[c] and Humberto González-Díaz[c,d]*

[a.] Department of Computer Science and Information Technologies, Faculty of Computer Science, University of A Coruña, Campus Elviña s/n, 15071, A Coruña, Spain.

[b.] IKERDATA S.L., ZITEK, University of Basque Country UPVEHU, Rectorate Building, 48940 Leioa, Spain.

[c.] Department of Organic and Inorganic Chemistry, Faculty of Science and Technology, University of The Basque Country (UPV/EHU), P.O. Box 644, 48080, Bilbao, Spain.

[d.] IKERBASQUE, Basque Foundation for Science, 48011, Bilbao, Spain.

TABLES

Table S1 Labels and Descriptions of the functional variables (V_k) used in the database.

Label	Description	Label	Description	Label	Description
V_1	Time of the catalyst treatment (min)	V_{18}	Power of the microwave (W) (Step 1)	V_{35}	Quantity of the nucleophile (mmol) (Step 2)
V_2	Minimum quantity of the metal absorbed in the catalyst (µg)	V_{19}	Dipole moment of the solvent 1 (D) (Step 1)	V_{36}	Quantity of the base (mmol) (Step 2)
V_3	Maximum quantity of the metal absorbed in the catalyst (µg)	V_{20}	Molecular weight of the solvent 1 (g/mol) (Step 1)	V_{37}	Base equivalent (Step 2)
V_4	Minimum quantity of the metal released from the catalyst (µg)	V_{21}	Density of the solvent 1 (g/mL) (Step 1)	V_{38}	Oxidant equivalent (Step 2)
V_5	Maximum quantity of the metal released from the catalyst (µg)	V_{22}	Volume of the solvent 1 (mL) (Step 1)	V_{39}	Quantity of the oxidant (mol) (Step 2)
V_6	1st dimension of the support (mm)	V_{23}	Solvent 1 equivalent (Step 1)	V_{40}	Additive equivalent (Step 2)
V_7	2nd dimension of the support (mm)	V_{24}	Molecular weight of the solvent 2 (g/mol) (Step 1)	V_{41}	Quantity of the additive (mmol) (Step 2)
V_8	Quantity of the electrophile (mmol)	V_{25}	Density of the solvent 1 (g/mL) (Step 1)	V_{42}	Power of the microwave (W) (Step 2)
V_9	Nucleophile equivalent (Step 1) (Step 1)	V_{26}	Volume of the solvent 2 (mL) (Step 1)	V_{43}	Volume of the solvent 1 (Step 2) (mL)
V_{10}	Quantity of the nucleophile (mmol) (Step 1)	V_{27}	Solvent 2 equivalent (Step 1)	V_{44}	Dipole moment of the solvent 1 (D) (Step 2)
V_{11}	pKa of the base	V_{28}	Dipole moment of the solvent 2 (D) (Step 1)	V_{45}	Volume of the solvent 2 (mL) (Step 2)
V_{12}	Base equivalent (Step 1)	V_{29}	Total dipole moment (Step 1)	V_{46}	Dipole moment of the solvent 2 (D) (Step 2)
V_{13}	Quantity of the base (mmol) (Step 1)	V_{30}	Temperature (ºC) (Step 1)	V_{47}	Total dipole moment (Step 1)
V_{14}	Oxidant equivalent (Step 1)	V_{31}	Time (h) (Step 1)	V_{48}	Temperature (ºC) (Step 2)
V_{15}	Quantity of the oxidant (mol) (Step 1)	V_{32}	Was the reaction mixed? Y/N (Step 1)	V_{49}	Time (h) (Step 2)
V_{16}	Additive equivalent (Step 1)	V_{33}	Minimum times the catalyst was reused	V_{50}	Was the reaction mixed? Y/N (Step 1)
V_{17}	Quantity of the additive (mmol) (Step 1)	V_{34}	Nucleophile equivalent (Step 2)		

Table S2 Labels and descriptions of the Molecular descriptors (D_k) used in the database.

Label	Description	Label	Description	Label	Description
D_1	Size of the catalyst (nm)	D_{11}	Squared Ghose-Crippen octanol-water partition coefficient (logP^2) of the electrophile	D_{21}	Ghose-Crippen octanol-water partition coefficient (logP)
D_2	Van der Waals radius of the catalyst's metal (pm)	D_{12}	Number of substituted benzene C(sp^2) of the electrophile	D_{22}	Squared Ghose-Crippen octanol-water partition coefficient (logP^2) of the nucleophile
D_3	Electronegativity of the catalyst's metal	D_{13}	Number of halogen atoms of the electrophile	D_{23}	Number of substituted benzene C(sp^2) of the nucleophile
D_4	Sum of atomic van der Waals volumes (scaled on Carbon atom) of the electrophile	D_{14}	Number of 6-membered rings of the electrophile	D_{24}	Number of Boron atoms of the nucleophile
D_5	Sum of atomic Sanderson electronegativities (scaled on Carbon atom) of the electrophile	D_{15}	Sum of atomic van der Waals volumes (scaled on Carbon atom) of the nucleophile	D_{25}	Number of 6-membered rings of the nucleophile
D_6	Sum of atomic polarizabilities (scaled on Carbon atom) of the electrophile	D_{16}	Sum of atomic Sanderson electronegativities (scaled on Carbon atom) of the nucleophile	D_{26}	Sum of atomic van der Waals volumes (scaled on Carbon atom) of the base
D_7	Sum of Kier-Hall electrotopological states of the electrophile	D_{17}	Sum of atomic polarizabilities (scaled on Carbon atom) of the nucleophile	D_{27}	Sum of atomic polarizabilities (scaled on Carbon atom) of the base
D_8	Moriguchi octanol-water partition coefficient (logP) of the electrophile	D_{18}	Sum of Kier-Hall electrotopological states of the nucleophile	D_{28}	Number of substituted benzene C(sp^2) of the product
D_9	Squared Moriguchi octanol-water partition coefficient (logP^2) of the electrophile	D_{19}	Moriguchi octanol-water partition coefficient (logP) of the nucleophile	D_{29}	Number of 6-membered rings of the product
D_{10}	Ghose-Crippen octanol-water partition coefficient (logP)	D_{20}	Squarded Moriguchi octanol-water partition coefficient (logP^2) of the nucleophile		

Table S3 Description and labels of the calculated combined values.

Label	Description	Label	Description
$D_2 \cdot V_2$	Cat. Met Van der Waals radius(pm)·Cat. Met ads. min qty(µg)	$D_{16} \cdot (V_{10} + V_{35})$	Se(Nucleophile)·Nucleophile(mmol)
$D_4 \cdot V_8$	Sv(Electrophile)·Electrophile(mmol)	$D_{17} \cdot (V_{10} + V_{35})$	Sp(Nucleophile)·Nucleophile(mmol)
$D_5 \cdot V_8$	Se(Electrophile)·Electrophile(mmol)	$D_{18} \cdot (V_{10} + V_{35})$	Ss(Nucleophile)·Nucleophile(mmol)
$D_6 \cdot V_8$	Sp(Electrophile)·Electrophile(mmol)	$D_{19} \cdot (V_{10} + V_{35})$	MLOGP(Nucleophile)·Nucleophile(mmol)
$D_7 \cdot V_8$	Ss(Electrophile)·Electrophile(mmol)	$D_{20} \cdot (V_{10} + V_{35})$	MLOGP2(Nucleophile)·Nucleophile(mmol)
$D_8 \cdot V_8$	MLOGP(Electrophile)·Electrophile(mmol)	$D_{21} \cdot (V_{10} + V_{35})$	ALOGP(Nucleophile)·Nucleophile(mmol)
$D_9 \cdot V_8$	MLOGP2(Electrophile)·Electrophile(mmol)	$D_{22} \cdot (V_{10} + V_{35})$	ALOGP2(Nucleophile)·Nucleophile(mmol)
$D_{10} \cdot V_8$	ALOGP(Electrophile)·Electrophile(mmol)	$V_{11} \cdot V_{13}$	pKa Base·Base(mmol)
$D_{11} \cdot V_8$	ALOGP2(Electrophile)·Electrophile(mmol)	$V_{19} \cdot V_{22}$	Solvent dipole mom(D)·V Solvent (Sol1)(mL)
$D_{15} \cdot (V_{10} + V_{35})$	Sv(Nucleophile)·Nucleophile(mmol)	$V_{29} \cdot (V_{22} + V_{27})$	Solvent dipole mom Tot(D)·V Solvent Tot(mL)

Table S4 Labels and descriptions of the condition variables (c_j) used in the database.

Label	Description	Label	Description	Label	Description
c_1	Characteristics of the support of the catalyst	c_4	Generation of the synthesis of the SAM/SGM	c_7	Reaction type
c_2	Form of the metal in the support	c_5	Catalyst metal	c_8	Yield detection
c_3	Treatment/Procedure of the catalyst support	c_6	Time of reuse of the catalyst	c_9	Step where the nucleophile was added

Table S5 Statistical results of the MLR models for each IFPTML partition.

PTML partition table	Procedure	Variables			Multiple R	Multiple R²	*p*-value	Std.Err. of Estimate
1[a]	FWS	• $\Delta V_1(c_I)$<br>• $\Delta V_{14}(c_{III})$<br>• $\Delta V_{17}(c_{III})$	• $\Delta V_{30}(c_{III})$<br>• $\Delta V_{33}(c_{III})$<br>• $\Delta V_{44}(c_{III})$	• $\Delta D_5(c_I)$<br>• $\Delta D_{26}(c_I)$<br>• $\Delta D_{29}(c_{III})$	0.8397	0.7051	< 0.05	14.7105
	FWS + EGS	• $\Delta[D_{16}\cdot(V_{10}+V_{35})](c_I)$<br>• $\Delta[V_{11}\cdot V_{13}](c_{III})$<br>• $\Delta[V_{29}\cdot(V_{22}+V_{26})](c_{III})$	• $\Delta[D_4\cdot V_8](c_I)$<br>• $\Delta[D_2\cdot V_2](c_I)$<br>• $\Delta V_{30}(c_{II})$	• $\Delta V_{31}(c_I)$<br>• $\Delta V_{33}(c_I)$	0.8210	0.6740	< 0.05	15.4089
2[a]	FWS	• $\Delta V_1(c_I)$<br>• $\Delta V_{14}(c_{III})$	• $\Delta V_{18}(c_{III})$<br>• $\Delta V_{30}(c_{III})$		0.8265	0.6831	< 0.05	15.2147
	FWS + EGS	• $\Delta[D_2\cdot V_2](c_I)$<br>• $\Delta[D_5\cdot V_8](c_I)$<br>• $\Delta[D_{16}\cdot\cdot(V_{10}+V_{35})](c_{II})$<br>• $\Delta[V_{29}\cdot(V_{22}+V_{26})](c_{II})$	• $\Delta[V_{11}\cdot V_{13}](c_{II})$<br>• $\Delta V_1(c_I)$<br>• $\Delta V_{14}(c_{III})$<br>• $\Delta V_{18}(c_{II})$	• $\Delta V_{30}(c_{III})$<br>• $\Delta V_{31}(c_I)$<br>• $\Delta V_{33}(c_I)$	0.8333	0.6944	< 0.05	14.9894
3[a]	FWS	• $\Delta V_1(c_I)$<br>• $\Delta V_{14}(c_{II})$	• $\Delta V_{18}(c_I)$<br>• $\Delta V_{30}(c_{II})$	• $\Delta V_{33}(c_I)$<br>• $\Delta D_{24}(c_I)$	0.8371	0.7007	< 0.05	14.7995
	FWS + EGS	• $\Delta[D_{15}\cdot(V_{10}+V_{35})](c_{II})$<br>• $\Delta[V_{11}\cdot V_{13}](c_{II})$<br>• $\Delta[V_{29}\cdot(V_{22}+V_{26})](c_{II})$	• $\Delta[D_6\cdot V_8](c_{II})$<br>• $\Delta[D_2\cdot V_2](c_I)$<br>• $\Delta V_{30}(c_{II})$	• $\Delta V_{31}(c_I)$<br>• $\Delta V_{33}(c_I)$	0.8177	0.6686	< 0.05	15.5863
4[a]	FWS	• $\Delta[D_{16}\cdot(V_{10}+V_{35})](c_I)$<br>• $\Delta V_{12}(c_I)$<br>• $\Delta V_{13}(c_I)$	• $\Delta V_{14}(c_I)$<br>• $\Delta V_{20}(c_I)$<br>• $\Delta V_{27}(c_I)$	• $\Delta V_{31}(c_I)$<br>• $\Delta V_{33}(c_I)$<br>• $\Delta D_1(c_I)$	0.8373	0.7012	< 0.05	14.8087
	FWS + EGS	• $\Delta[D_{17}\cdot(V_{10}+V_{35})](c_I)$<br>• $\Delta[V_{11}\cdot V_{13}](c_I)$<br>• $\Delta[V_{29}\cdot(V_{22}+V_{26})](c_I)$	• $\Delta[D_6\cdot V_8](c_I)$<br>• $\Delta[D_2\cdot V_2](c_I)$<br>• $\Delta V_{30}(c_I)$	• $\Delta V_{31}(c_I)$<br>• $\Delta V_{33}(c_I)$	0.8157	0.6653	< 0.05	15.6647
1[b]	FWS	• $\Delta V_{30}(c_{III})$	• $\Delta V_{36}(c_I)$		0.8205	0.6732	< 0.05	15.3634
	FWS + EGS	• $\Delta[D_{16}\cdot V_{10}](c_I)$<br>• $\Delta[V_{11}\cdot V_{13}](c_{III})$<br>• $\Delta V_{29}\cdot(\Delta V_{22}+\Delta V_{26})(c_{III})$	• $\Delta[D_2\cdot V_2](c_I)$<br>• $\Delta[D_4\cdot V_8](c_I)$<br>• $\Delta V_{30}(c_{III})$	• $\Delta V_{31}(c_I)$<br>• $\Delta V_{33}(c_I)$	0.8181	0.6694	< 0.05	15.5775
2[b]	FWS	• $\Delta[D_5\cdot V_8](c_I)$<br>• $\Delta V_8(c_{II})$<br>• $\Delta V_{10}(c_I)$	• $\Delta V_{15}(c_{II})$<br>• $\Delta V_{18}(c_{II})$<br>• $\Delta V_{23}(c_{II})$	• $\Delta V_{21}(c_{III})$<br>• $\Delta V_{30}(c_{III})$<br>• $\Delta D_{26}(c_{III})$	0.8437	0.7118	< 0.05	14.5530
	FWS + EGS	• $\Delta[D_2\cdot V_2](c_I)$<br>• $\Delta[D_5\cdot V_8](c_I)$<br>• $\Delta[D_{16}\cdot(V_{10}+V_{35})](c_I)$	• $\Delta V_{15}(c_{II})$<br>• $\Delta V_{18}(c_{II})$<br>• $\Delta V_{23}(c_{II})$	• $\Delta V_{31}(c_I)$<br>• $\Delta V_{31}(c_{III})$<br>• $\Delta V_{33}(c_I)$	0.8204	0.6730	< 0.05	15.5010
3[b]	FWS	• $\Delta[V_{19}\cdot V_{22}](c_I)$<br>• $\Delta[D_4\cdot V_8](c_I)$<br>• $\Delta V_8(c_I)$	• $\Delta V_8(c_{II})$<br>• $\Delta V_{15}(c_I)$<br>• $\Delta V_{18}(c_I)$	• $\Delta V_{22}(c_{II})$<br>• $\Delta V_{30}(c_{II})$<br>• $\Delta D_{26}(c_{II})$	0.8440	0.7124	< 0.05	14.5367
	FWS + EGS	• $\Delta[V_{11}\cdot V_{13}](c_{II})$<br>• $\Delta[V_{29}\cdot(V_{22}+V_{26})](c_{II})$<br>• $\Delta[D_{15}\cdot(V_{10}+V_{35})](c_{II})$	• $\Delta[D_2\cdot V_2](c_I)$<br>• $\Delta[D_6\cdot V_8](c_{II})$<br>• $\Delta V_{30}(c_{II})$	• $\Delta V_{31}(c_I)$<br>• $\Delta V_{33}(c_I)$	0.8181	0.6693	< 0.05	15.5787
4[b]	FWS	• $\Delta V_{12}(c_I)$	• $\Delta V_{25}(c_I)$		0.8248	0.6803	< 0.05	15.2639
	FWS + EGS	• $\Delta[D_5\cdot V_8](c_I)$<br>• $\Delta[V_{29}\cdot(V_{22}+V_{26})](c_I)$<br>• $\Delta[D_{17}\cdot(V_{10}+V_{35})](c_I)$	• $\Delta[D_2\cdot V_2](c_I)$<br>• $\Delta V_{12}(c_I)$<br>• $\Delta V_{30}(c_I)$	• $\Delta V_{31}(c_I)$<br>• $\Delta V_{33}(c_I)$	0.8289	0.6870	< 0.05	15.1549

[a]$n_{train} = 1134$. [b]$n_{train} = 851$

Table S6 Statistical results of the MLR models for each IFPTML partition without the double carbonylation set (n_{train} = 790).

PTML partition table	Procedure	Variables			Multiple R	Multiple R²	*p*-value	Std.Err. of Estimate
1	FWS	• $\Delta V_{30}(c_{III})$<br>• $\Delta V_{8}(c_{III})$	• $\Delta V_{24}(c_{I})$		0.8732	0.7625	< 0.05	11.8014
	FWS + EGS	• $\Delta[D_4 \cdot V_8](c_{II})$<br>• $\Delta[D_{16} \cdot (V_{10} + V_{35})](c_{III})$<br>• $\Delta V_{29} \cdot (\Delta V_{22} + \Delta V_{26})(c_{III})$	• $\Delta[D_2 \cdot V_2](c_I)$<br>• $\Delta[V_{11} \cdot V_{13}](c_{III})$<br>• $\Delta V_{30}(c_{III})$	• $\Delta V_{31}(c_I)$<br>• $\Delta V_{33}(c_I)$	0.8601	0.7398	< 0.05	12.4799
2	FWS	• $\Delta[D_{15} \cdot (V_{10} + V_{35})](c_I)$<br>• $\Delta V_2(c_{III})$<br>• $\Delta V_8(c_I)$	• $\Delta V_8(c_{III})$<br>• $\Delta V_{15}(c_I)$<br>• $\Delta V_{18}(c_{II})$	• $\Delta V_{29}(c_{II}$<br>• $\Delta V_{30}(c_{III}$<br>• $\Delta D_{26}(c_{III})$	0.8985	0.8072	< 0.05	10.7476
	FWS + EGS	• $\Delta[V_{29} \cdot (V_{22} + V_{26})](c_I)$<br>• $\Delta[D_{15} \cdot (V_{10} + V_{35})](c_I)$<br>• $\Delta[D_{11} \cdot V_{13}](c_{III})$	• $\Delta[D_6 \cdot V_8](c_{III})$<br>• $\Delta[D_2 \cdot V_2](c_I)$<br>• $\Delta V_{30}(c_{II})$	• $\Delta V_{31}(c_I)$<br>• $\Delta V_{33}(c_I)$	0.8496	0.7218	< 0.05	14.9894
3	FWS	• $\Delta V_1(c_I)$<br>• $\Delta V_8(c_I)$<br>• $\Delta V_{12}(c_{II})$	• $\Delta V_{15}(c_I)$<br>• $\Delta V_{18}(c_I)$<br>• $\Delta V_{29}(c_I)$	• $\Delta V_{29}(c_{II}$<br>• $\Delta V_{30}(c_I)$<br>• $\Delta D_{26}(c_{II})$	0.8954	0.8017	< 0.05	10.9022
	FWS + EGS	• $\Delta[D_{15} \cdot (V_{10} + V_{35})](c_{II})$<br>• $\Delta[V_{11} \cdot V_{13}](c_{II})$<br>• $\Delta[V_{29} \cdot (V_{22} + V_{26})](c_{II})$	• $\Delta[D_6 \cdot V_8](c_{II})$<br>• $\Delta V_{30}(c_{II})$<br>• $\Delta[D_2 \cdot V_2](c_I)$	• $\Delta V_{31}(c_I)$<br>• $\Delta V_{33}(c_I)$	0.8604	0.7404	< 0.05	12.4653
4	FWS	• $\Delta V_1(c_I)$<br>• $\Delta V_{12}(c_I)$<br>• $\Delta V_{13}(c_I)$	• $\Delta V_{15}(c_I)$<br>• $\Delta V_{19}(c_I)$<br>• $\Delta V_{25}(c_I)$	• $\Delta D_{21}(c_I)$<br>• $\Delta D_{13}(c_I)$<br>• $\Delta D_{27}(c_I)$	0.8952	0.8013	< 0.05	10.9113
	FWS + EGS	• $\Delta[D_2 \cdot V_2](c_I)$<br>• $\Delta[V_{29} \cdot (V_{22} + V_{26})](c_I)$<br>• $\Delta[D_{17} \cdot (V_{10} + V_{35})](c_I)$	• $\Delta[D_6 \cdot V_8](c_I)$<br>• $\Delta[V_{11} \cdot V_{13}](c_I)$<br>• $\Delta V_{30}(c_I)$	• $\Delta V_{31}(c_I)$<br>• $\Delta V_{33}(c_I)$	0.8723	0.7609	< 0.05	11.9625

Table S7 Results of the ANN models with all the variables (PTML 3).

Profile	Training/ Members[c]	Inputs	H (1)	H (2)	R (Train)	R (Test)
LNN[a]<br>174:174-1:1	PI	174	0	0	0.6842	0.6345
MLP[a]<br>83:83-22-1:1	BP100<br>CG20<br>CG255b	83	22	0	0.8766	0.8131
RBF[a]<br>30:30-43-1:11	KM<br>KN<br>PI	30	43	0	0.8520	0.8161
GRNN[a]<br>179:179-851-2-1:11:11	SS	179	851	2	0.8759	0.7813
LNN[b]<br>175:175-1:1	PI	175	0	0	0.7464	0.6998
MLP[b]<br>97:97-50-28-1:1	BP10<br>CG2<br>CG641b	97	50	28	0.9587	0.8863
RBF[b]<br>33:33-39-1:1	KM<br>KN<br>PI	33	39	0	0.9046	0.8916
GRNN[b]<br>179:179-790-2-1:1	SS	179	790	2	0.9295	0.8713

[a]Models with the complete dataset. [b] Models without the double carbonylation subset. [c] The codes are: BP = Back Propagation, CG = Conjugate Gradient Descent, SS = Sub Sample, KM = K-Means (Centre Assignment), KN = K-Nearest Neighbour (Deviation Assignment), PI = Pseudo-Invert (Linear Least Squares Optimization).

Table S8 Training and test performance metrics of the MLP (9:9-20-18-1:1) classification model with the EGS variables (PTML 3).

Dataset	Accuracy	Precision	Recall	F1
Training set	0.9397	0.9755	0.9432	0.9591
Test set	0.9340	0.9712	0.9395	0.9551

FIGURES

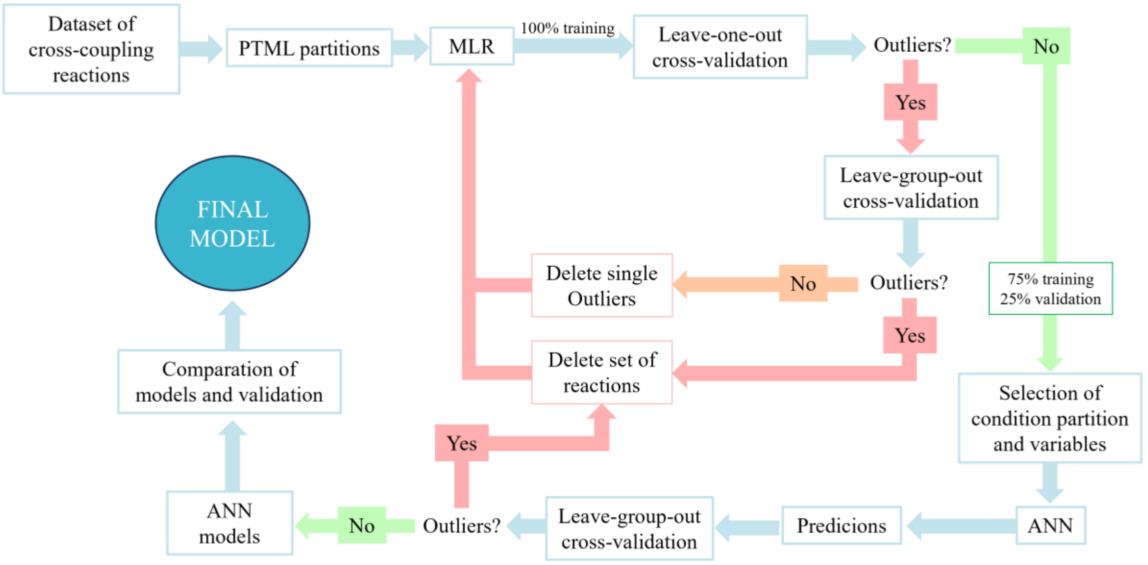

Figure SI1 The workflow used to build MLR and ANN models.

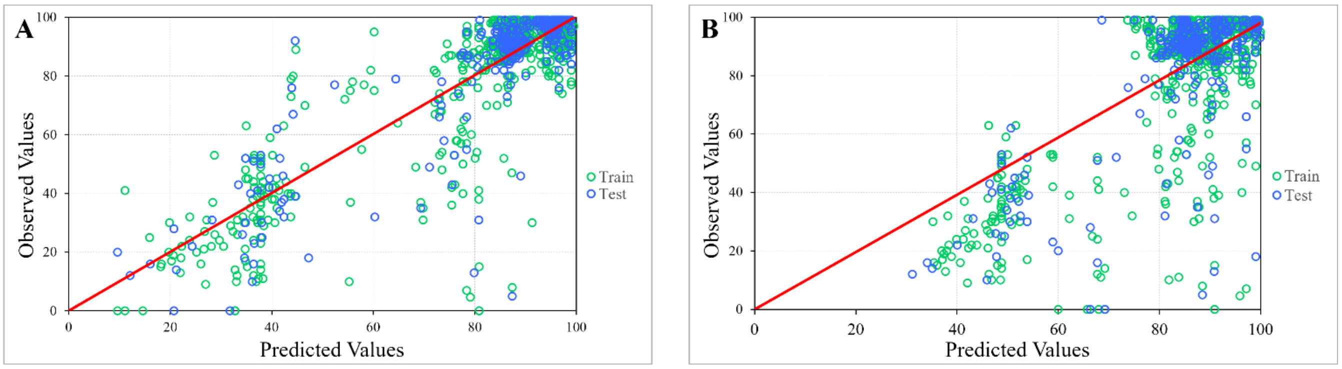

Figure SI2 Observed values vs predicted values of training and validation data of PTML 3 (A) and PTML 4 (B) condition partition

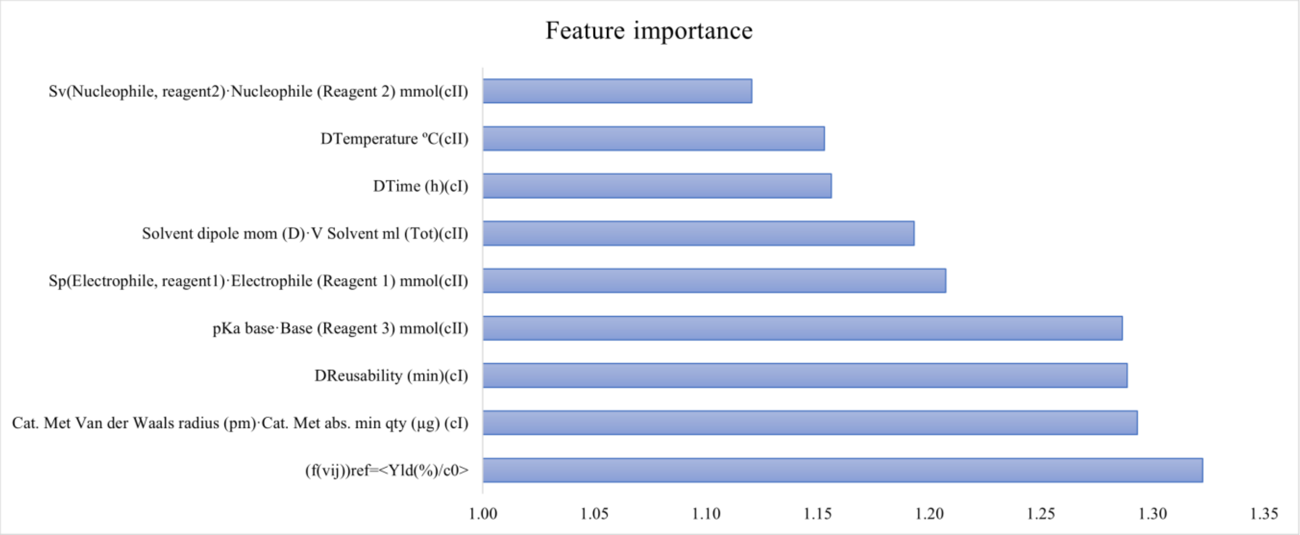

Figure SI3 Feature importance ranking for the MLP (9:9-20-18-1:1) classification model.
